# S3QELs protect against diet‐induced intestinal barrier dysfunction

**DOI:** 10.1111/acel.13476

**Published:** 2021-09-14

**Authors:** Mark A. Watson, Blaine Pattavina, Tyler A. U. Hilsabeck, Jose Lopez‐Dominguez, Pankaj Kapahi, Martin D. Brand

**Affiliations:** ^1^ The Buck Institute for Research on Aging Novato California USA

**Keywords:** aging, complex III, diet, drosophila, intestine, intestinal permeability, leaky gut, metabolism, mitochondria, oxidative stress, superoxide

## Abstract

The underlying causes of aging remain elusive, but may include decreased intestinal homeostasis followed by disruption of the intestinal barrier, which can be mimicked by nutrient‐rich diets. S3QELs are small‐molecule suppressors of site III_Qo_ electron leak; they suppress superoxide generation at complex III of the mitochondrial electron transport chain without inhibiting oxidative phosphorylation. Here we show that feeding different S3QELs to *Drosophila* on a high‐nutrient diet protects against greater intestinal permeability, greater enterocyte apoptotic cell number, and shorter median lifespan. Hif‐1α knockdown in enterocytes also protects, and blunts any further protection by S3QELs. Feeding S3QELs to mice on a high‐fat diet also protects against the diet‐induced increase in intestinal permeability. Our results demonstrate by inference of S3QEL use that superoxide produced by complex III in enterocytes contributes to diet‐induced intestinal barrier disruption in both flies and mice.

## Graphical abstract

Animals fed a high‐nutrient diet exhibit decreased intestinal integrity over time, which coincides with a decreased lifespan. S3QELs inhibit superoxide production specifically from site III_Qo_ of complex III in mitochondria. Treatment of animals on a high‐nutrient diet with S3QELs increases intestinal integrity resulting in an increased lifespan potentially by decreasing ER stress and Hif‐1α signaling.

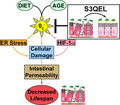

## 1 INTRODUCTION

The intestinal epithelium has several barriers, consisting of a mucous layer, tight junctions between cells, and a substantial set of resident immune cells, to protect the host from pathogens and toxins in the gut lumen (Peterson & Artis, 2014; Vighi et al., 2008). Disruption of the intestinal epithelial barrier permits the passage of these pathogens and toxins, which can initiate and exacerbate disease and possibly aging (Doig et al., 1998; Fink & Delude, 2005; Harris et al., 1992). Understanding and preventing the underlying causes of intestinal barrier decline could help prevent disease and possibly slow aging (Farhadi et al., 2003; Fasano & Shea‐Donohue, 2005; König et al., 2016; Odenwald & Turner, 2017). Oxidative stress has been argued to be an important driver of aging and age‐related pathologies, including intestinal barrier dysfunction (Hale et al., 2012; Liguori et al., 2018; Rera et al., 2012; Tian et al., 2017; Wang et al., 2014). However, the sources of oxidative stress and their relative importance in a given pathology remain unclear. In the present study, we investigated whether mitochondrial superoxide generated by complex III of the mitochondrial electron transport chain is a cause of intestinal barrier disruption, using *Drosophila* and mice fed high‐nutrient diets as models of accelerated metabolic disease and aging.

**FIGURE 1:**
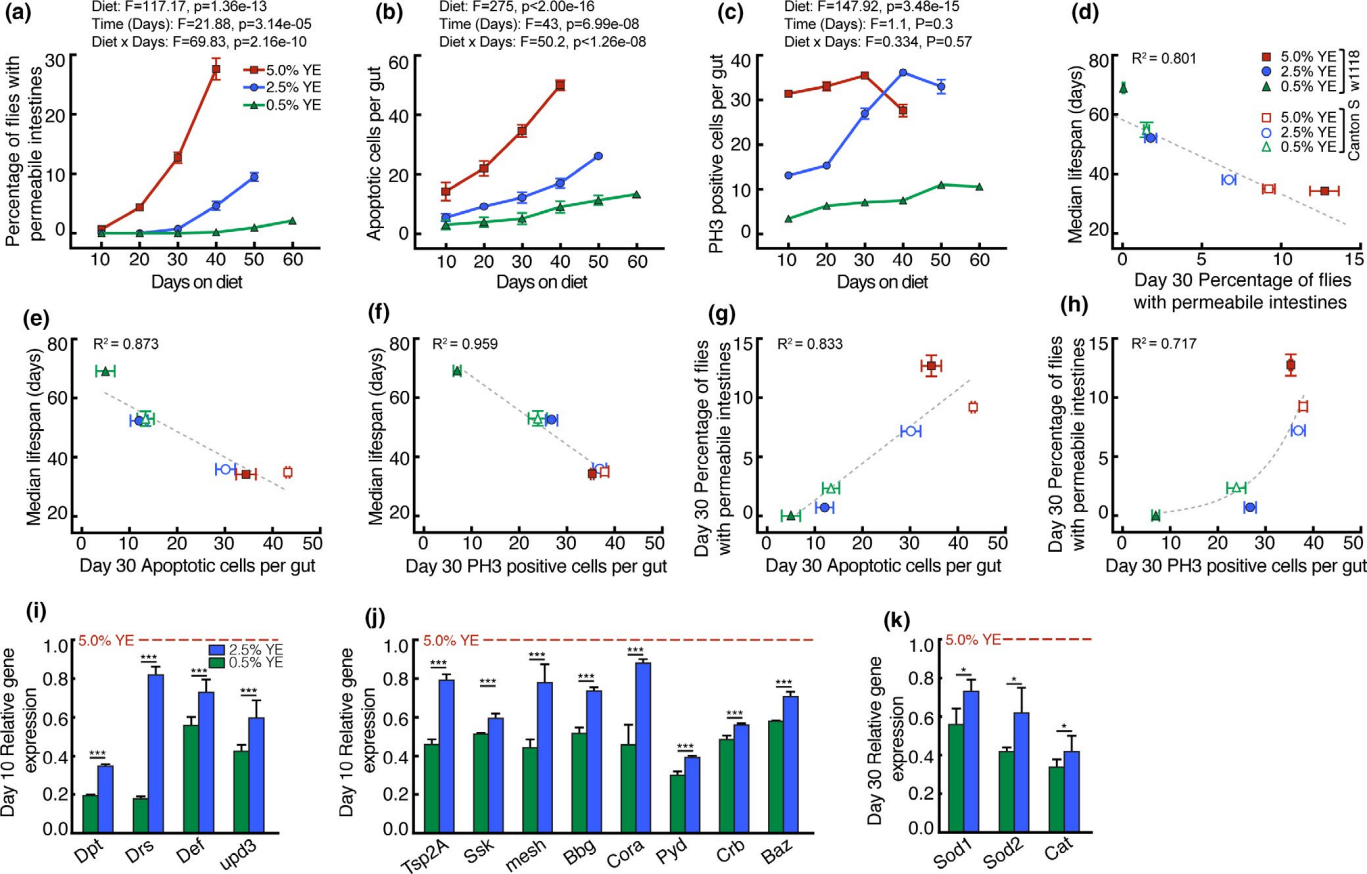
Effects of diet on time‐dependent intestinal parameters and lifespan in *Drosophila*. After eclosion *Drosophila* were raised for five days on standard yeast medium then switched on day 5 to diets containing 0.5–5% (w/v) yeast extract (YE). Effects of YE% on (a) intestinal permeability in *w^1118^* flies, (b) number of intestinal apoptotic cells, and (c) number of intestinal proliferating stem cells measured using phosphohistone H3 (PH3). (d‐h) Relationships between median lifespan, intestinal permeability, intestinal apoptotic number and intestinal PH3‐positive cell number of *w^1118^* and *canton S* flies. Lines were fit using either linear regression (d‐g) or exponential growth (h). (i‐k) Effect of YE% on intestinal gene expression normalized to *Rp49* then expressed as fold change relative to 5% YE. Panels show (i) inflammatory and damage markers (j) intestinal tight junction genes and (k) antioxidant genes. Data are means ± SEM of *n* = 3 biological replicates each using 200 flies (a), or 12 (b, c) or 15 (i‐k) dissected intestines. *p < 0.05, ***p < 0.0001 by ANCOVA (a‐c) or one‐way ANOVA with Tukey's post‐test with Dunnett's multiple comparisons test (i‐k). Lines in (d‐g) were fit using linear regression, and the line in (h) was fit using exponential regression. *F*, *F*‐value; *p*, *p*‐value

In many species, restricted or *ad*‐*libitum* feeding impacts both healthspan and lifespan. In *Drosophila*, decreasing the amount of dietary protein (in the form of yeast extract, YE) below the conventional 2.5% is known to increase median lifespan and decrease intestinal permeability, whereas increasing dietary YE content decreases median lifespan and increases intestinal permeability. Work from the laboratories of Hansen (Gelino et al., 2016), Walker (Rera et al., 2012), and Patridge (Regan et al., 2016) has shown that dietary restriction can prominently modulate lifespan and intestinal barrier dysfunction in both worm and flies. We confirmed this in the two *Drosophila* strains (*w^1118^* and *Canton S*) used here (Figure S1A,B). We measured intestinal permeability of flies using the “smurf assay” which involves feeding a blue food dye that is normally not absorbed; flies with permeable intestinal epithelium become blue (Rera et al., 2012). The percentage of blue flies was higher at greater YE% in both *w^1118^* (Figure 1a) and *Canton S* flies (Figure S1C). Analysis by ANCOVA affirmed significant effects of diet and days on diet on intestinal permeability, and a significant interaction of both variables. Further analysis revealed that median lifespan was decreased in flies of either strain when the incidence of intestinal permeability was enhanced by greater dietary YE% (Figure 1d), suggesting the possibility that intestinal permeability influences lifespan.

There is a critical balance between cell proliferation and apoptosis in the *Drosophila* intestine, and tipping this balance can be detrimental (Akagi et al., 2018; Liang et al., 2017). Enterocyte damage can cause apoptosis and therefore trigger proliferation of intestinal cells for repair and maintenance of tissue integrity (Amcheslavsky et al., 2009; Ohlstein & Spradling, 2006). Feeding higher YE% to *w^1118^* and *Canton S* flies increased both the number of apoptotic cells and the number of proliferating (PH3‐positive) cells per intestine (Figure 1b,c; Figure S1D,E), supporting this view. Analysis by ANCOVA affirmed significant effects of diet and days on diet on intestinal apoptosis, and a significant interaction of both variables. However, only diet had a significant effect on PH3‐positive intestinal cell number. Apoptotic and PH3‐positive intestinal cell number correlated negatively with median lifespan (Figure 1e,f) and positively with intestinal permeability (Figure 1g,h).

We confirmed an increase in intestinal permeability using known gene expression hallmarks of a disrupted intestinal barrier (Rera et al., 2012). Antimicrobial peptides (AMPs) are expressed in response to intestinal damage and infection, and the Upd3 cytokine is released upon enterocyte damage (Lucchetta & Ohlstein, 2012). The intestinal expression of the AMP genes *Dpt*, *Drs* and *Def*, and the *upd3* gene increased at higher YE% on day 10 (Figure 1i) and day 30 (Figure S1F) after the diet switch. The intestinal expression of septate junction genes (the equivalent of tight junctions in vertebrates) also increased at higher dietary YE% (Figure 1j; Figure S1G). Elevated expression of tight junction genes in flies fed a high‐nutrient diet suggests a response to epithelial tight junction damage that is related to an increase in intestinal permeability.

Next, we investigated the mechanism by which a rich diet decreased median lifespan and increased intestinal permeability. Increased oxidative stress is reported in intestines of animals fed high‐nutrient diets (Feillet‐Coudray et al., 2014; Lee et al., 2017; Paglialunga et al., 2015; Patel et al., 2007). We hypothesized that mitochondrial production of reactive oxygen species might drive increased intestinal permeability, a suggestion supported by the observation of elevated intestinal gene expression of superoxide dismutases (which dismutate superoxide to hydrogen peroxide) in the cytosol (*Sod1*) and mitochondria (*Sod2*), and of catalase (*Cat*), which dismutates hydrogen peroxide to water and oxygen, in flies fed higher YE% (Figure 1k).

The outer ubiquinone‐binding site of complex III of the mitochondrial electron transport chain (site III_Qo_) has the largest capacity of all mitochondrial sites to produce superoxide, which it delivers into both the mitochondrial matrix and the cytosol (Brand, 2016; St‐Pierre et al., 2002). S3QELs are specific small‐molecule Suppressors of site III_Qo_ Electron Leak that suppress superoxide generation at complex III of the mitochondrial electron transport chain without inhibiting normal electron flow or oxidative phosphorylation or having any other known cellular targets (Orr et al., 2015). S3QELs are a more specific way to evaluate the role of superoxide in a phenotype than conventional approaches of genetic knockdowns of superoxide‐producing respiratory complexes, which (by inhibiting electron transport) have deeply confounding pleiotropic effects, or directly measuring superoxide levels in vivo, using methods that are widely recognized to be plagued by non‐selectivity and/or measurement artefacts (Murphy et al., 2011).

To test whether superoxide produced by site III_Qo_ contributes to intestinal permeability, flies fed a 5% YE diet were also fed S3QELs. We tested three S3QELs: S3QEL1.2, S3QEL2.2, and S3QEL3 (Figure S10). These act as their own internal control as all three structurally different S3QELs should give the same response if their effect is on‐target, but different responses if it is off‐target. We previously showed that S3QELs suppress superoxide/hydrogen peroxide production from site III_Qo_ by mitochondria isolated from *Drosophila* (Brand et al., 2016).

**FIGURE 2:**
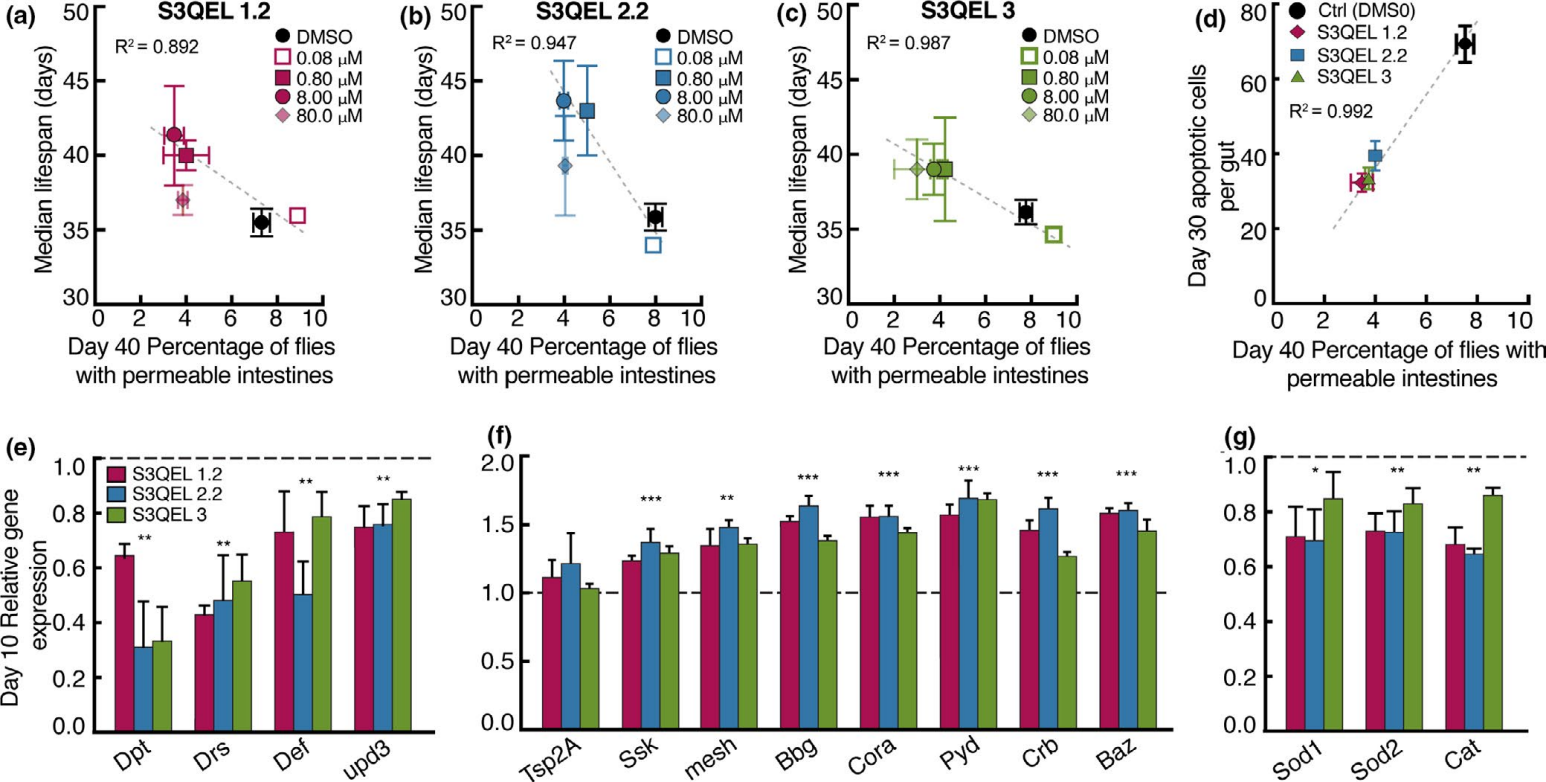
Effects of S3QELs on time‐dependent intestinal parameters and lifespan in *Drosophila* on 5% YE. After eclosion *w^1118^ Drosophila* were raised for five days on standard yeast medium then switched on day 5 to diets containing 5% YE with either S3QEL or DMSO vehicle. (a‐d) Relationships between median lifespan (see Figure S4 for lifespan curves), intestinal permeability (see Figure S2 for longitudinal intestinal permeability), and intestinal apoptotic number between S3QEL‐ and DMSO‐treated flies (Data using 8 μM of S3QELs is shown in figure d). Lines in (a‐d) were fit using linear regression. (e‐g) Effect of 8 μM of each S3QEL on intestinal gene expression normalized to *Rp49* then expressed as fold change relative to DMSO vehicle. Panels show (e) inflammatory and damage markers (f) intestinal tight junction genes and (g) antioxidant genes. Data are means ± SEM of *n* = 3 biological replicates each using 200 flies for median lifespan and intestinal permeability (a‐c), 12 intestines for intestinal apoptotic number (d) or 15 intestines (e‐g). *p < 0.05, **p < 0.001, ***p < 0.0001 by one‐way ANOVA with Tukey's post‐test (e‐g)

The incidence of intestinal permeability in *w^1118^ Drosophila* fed a 5% YE diet was approximately halved by co‐feeding each of the three S3QELs (Figure S2). The S3QELs also decreased the number of apoptotic cells per intestine (Figure S3a,b show that 8 μM S3QELs halved apoptotic cell number at both day 10 and day 30), and increased median lifespan by 10–20% (Figure S4). Interestingly, S3QELs had no significant effect on the intestinal incidence of PH3‐positive cells (Figure S3C–F). Further analysis revealed that median lifespan was greater (Figure 2a–c) and apoptotic cell number was smaller (Figure 2d) when feeding S3QELs at concentrations that decreased the incidence of intestinal permeability (i.e., at concentrations greater than 0.08 μM). These data suggest that by inhibiting superoxide production from site III_Qo_, S3QELs decrease intestinal permeability, which in turn correlates with an increase in median lifespan (Figure 2a–c). S3QELs did not significantly lower food palatability (Figure S5A) or consumption (Figure S5B), providing no evidence for the possibility that they worked by causing caloric restriction. In contrast to the effects of S3QELs, feeding of S1QEL1.1, S1QEL1.2, or S1QEL2.2 (suppressors of site I_Q_ electron leak (Brand et al., 2016)) did not protect against induction of intestinal permeability (Figure S6). This is in contrast to our previous finding that both S1QELs and S3QELs protected against stem cell hyperplasia in flies treated with tunicamycin or genetically overexpressing the oncogene Ras^V12^ (Brand et al., 2016). Comparison of these findings illustrates that different biological perturbations can modulate different sites of mitochondrial superoxide production, and that S1QELs and S3QELs are valuable tools to address such differences (Watson et al., 2019). Note that S1QELs and S3QELs each suppress superoxide/hydrogen peroxide production from the appropriate site by mitochondria isolated from *Drosophila* (Brand et al., 2016). Feeding S3QELs decreased the expression of the intestinal damage and inflammatory gene markers (Figure 2e), supporting the conclusion of a decrease in intestinal permeability relative to 5% YE diet. Expression of the tight junction genes did not decrease; instead it increased (Figure 2f). Feeding S3QELs decreased the expression of the antioxidant genes (Figure 2g), consistent with a decrease in oxidative stress.

**FIGURE 3:**
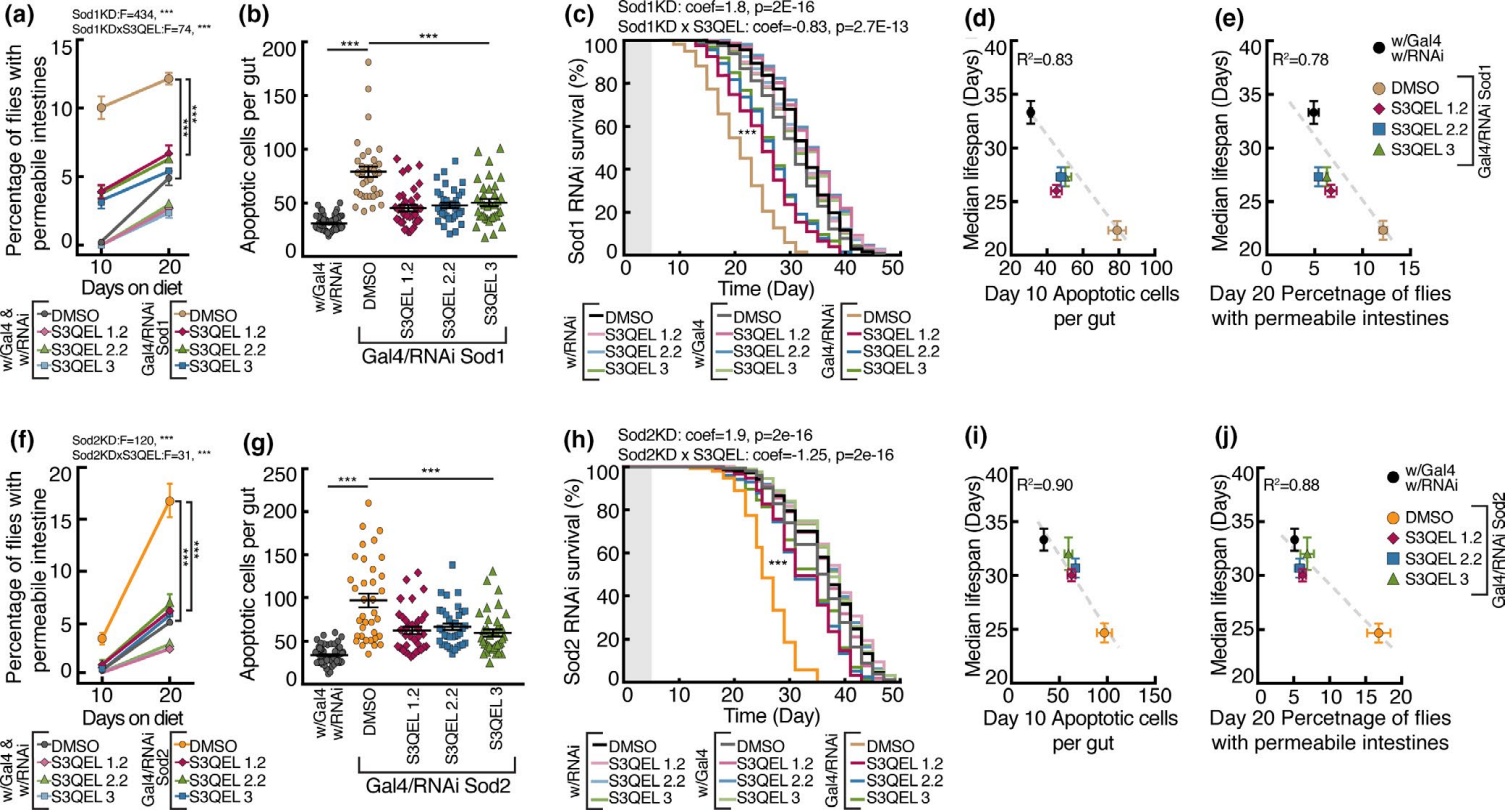
Effects of S3QELs in intestinal‐specific knockdown of superoxide dismutases (Sod1 and Sod2) in *Drosophila* reared on 5.0% YE diet. Intestinal enterocyte‐specific knockdown of cytosolic Sod1 or mitochondrial Sod2 was initiated 5 days after eclosion when flies at 18℃ were transferred to 5% YE with either 8 μM S3QEL or DMSO vehicle at 29℃. Effect of S3QELs on intestinal permeability (a, f), number of intestinal apoptotic cells (b, g), and lifespan (c, h), and relationships between median lifespan and intestinal permeability and intestinal apoptotic cell number (d, e, i, j) under Sod1 (a, b, c, d, e) and Sod2 (f, g, h, i, j) intestinal enterocyte knockdown. Data are means ± SEM of *n* = 3 biological replicates each using 200 flies (a, c, f, h), or 12 intestines (b, g). *p < 0.05, **p < 0.005, ***p < 0.0001 by three‐way ANOVA (a, f), or one‐way ANOVA with Dunnett's multiple comparisons test (b, g). Lifespan curves in (c, h) were analyzed using the Cox proportional hazards model. Shaded boxes in lifespan graphs (c‐h) indicate the 5‐day post‐eclosion period before flies were transferred to 5% YE. Lines in (d, e, i, j) were fit using linear regression. *F*, *F*‐value; *p*, *p*‐value; coef, coefficient

As proof of concept that S3QELs decrease mitochondrial superoxide production and can specifically work within gut enterocytes, we genetically lowered the antioxidant defenses of intestinal enterocytes. Using the NP1‐GAL4 driver, we used RNAi to knock down expression of *Sod1* and *Sod2* specifically in intestinal enterocytes to decrease either cytosolic (*Sod1*) or matrix superoxide removal (*Sod2*) and thereby increase superoxide levels in the two compartments in intestinal enterocytes. Figure 3 shows that each knockdown significantly increased the incidence of intestinal permeability and intestinal apoptotic cell number and decreased median lifespan, showing that raised superoxide levels just in enterocytes can drive these phenotypes. During either *Sod1* or *Sod2* knockdown, S3QELs protected against the induced intestinal permeability (Figure 3a,f), increase in intestinal apoptotic cell number (Figure 3b,g) and decrease in median lifespan (Figure 3c,h), improving the negative correlation between intestinal permeability, intestinal apoptotic cell number, and median lifespan caused by either *Sod1* or *Sod2* knockdown (Figure 3d,e,i,j). These results support the idea that S3QELs improve intestinal homeostasis and extend lifespan under a rich nutrient diet by decreasing superoxide production from complex III into both the cytosol (protection against *Sod1* knockdown) and matrix (protection against *Sod2* knockdown) specifically in enterocytes. Although the Sod knockdown experiments are a good way to manipulate superoxide concentrations independently in the matrix and cytosol to demonstrate their importance, we have not directly measured changes in superoxide concentration in vivo because of the unreliability of the probes that are available (Murphy et al., 2011); this is a limitation of our study.

*In vitro*, addition of a reductant such as succinate generates a semiquinone in complex III to drive superoxide production from site III_Qo_ (Brand, 2016). Previously we found that succinate concentration was elevated in flies reared on 5% YE compared to flies reared on 0.5% YE (Laye et al., 2015). Here we found that raising flies on 5% YE supplemented with 50 mM dimethyl succinate decreased median lifespan (Figure S7A) and increased intestinal permeability (Figure S7B) compared to 5% YE alone. This suggests that further elevation of succinate may exacerbate the 5% YE phenotypes by driving superoxide production from site III_Qo_.

We investigated how inhibiting superoxide production from site III_Qo_ with S3QELs ameliorates the effects of 5% YE diet other than perhaps preventing direct macromolecular damage. Superoxide generated from site III_Qo_ and succinate are both known to stabilize Hif‐1α (Bell et al., 2007; Brunelle et al., 2005; Guzy et al., 2005). Our previous work showed that S3QELs block the stabilization of Hif‐1α under hypoxic conditions *in vitro* (Orr et al., 2015). Utilizing the gene switch driver 5966(GS)‐GAL4 we explored whether Hif‐1α contributes to the decrease in lifespan and increased intestinal permeability of flies on 5% YE compared to 0.5% YE. Specific knockdown of Hif‐1α in enterocytes using RNAi resulted in an increase in lifespan (Figure S7C) and a substantial decrease in intestinal permeability (Figure S7D) in flies reared on 5% YE but not in flies reared on a 0.5% YE. Overexpression of Hif‐1α in enterocytes resulted in a further decrease in lifespan and increase in intestinal permeability in flies fed 5% YE (Figure S7E,F). Overexpression also resulted in a significant decrease in lifespan and an increase in intestinal permeability in flies fed 0.5% YE. These data suggest that elevated intestinal levels of Hif‐1α are detrimental and may drive some of the differences seen between 5% and 0.5 YE diet. Hif‐1α is already extensively associated with intestinal barrier dysfunction making it a plausible candidate target of S3QELs (Manresa & Taylor, 2017).

**FIGURE 4:**
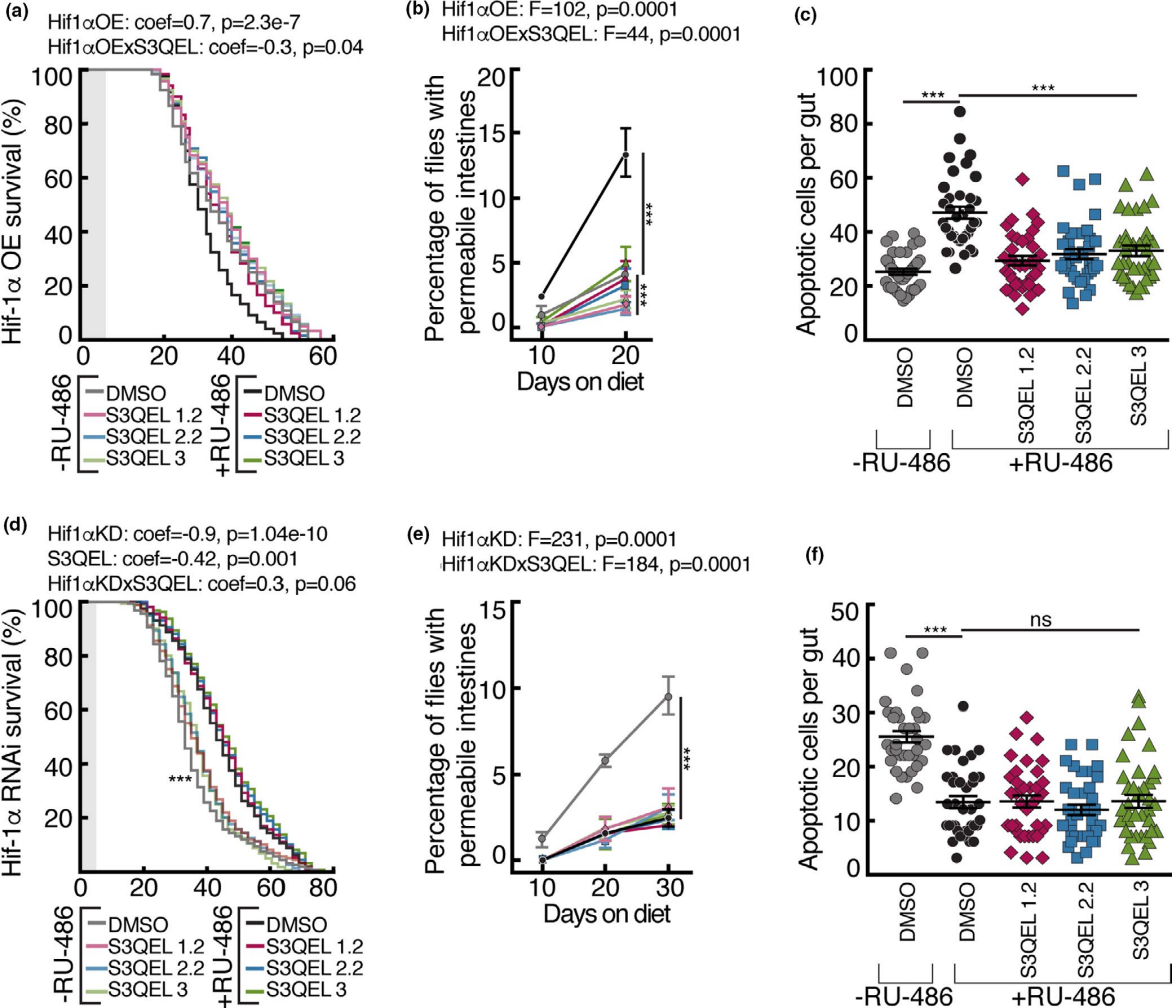
Effects of S3QELs in intestinal‐specific overexpression and knockdown of Hif‐1α in *Drosophila* reared on 5.0% YE diet. Intestinal enterocyte‐specific knockdown or overexpression of Hif‐1α was achieved using driver 5966(GS)‐GAL4. S3QELs were used at 8 µM. Effect of Hif‐1α overexpression in the presence of S3QELs on (a) lifespan, (b) intestinal permeability, and (c) number of intestinal apoptotic cells. Effect of Hif‐1α knockdown in the presence of S3QELs on (d) lifespan, (e) intestinal permeability, and (f) number of intestinal apoptotic cells. Data are means ± SEM of *n* = 3 biological replicates each using 140 flies (a, d), or 12 intestines (c, f). ***p < 0.0001 by either one‐way ANOVA with Dunnett's multiple comparisons test (c, f) or three‐way ANOVA (b, e). Lifespan curves in (a, d) were analyzed using the Cox proportional hazards model. Shaded boxes in lifespan graphs (a‐d) indicate the 5‐day post‐eclosion period before flies were transferred to 5% YE. coef, coefficient; *F*, *F*‐value; *p*, *p*‐value

To explore whether the effects of inhibition of superoxide production from site III_Qo_ are mediated by Hif‐1α in flies reared on 5% YE, we investigated the effects of S3QELs in flies with genetically altered levels of Hif‐1α in enterocytes. S3QELs protected more strongly against decreased lifespan and increased intestinal permeability in flies overexpressing Hif‐1α than in controls (Figure 4a,b). S3QEL treatment also protected against increased intestinal apoptotic cells (Figure 4c). However, in flies in which Hif‐1α was knocked down, S3QEL treatment did not further increase lifespan or decrease the number of intestinal apoptotic cells any further than DMSO‐treated knockdown flies (Figure 4f). S3QEL treatment had a small but significant interaction with Hif‐1α knockdown (Figure 4d,e). These data suggest that the effects of S3QELs are blunted when Hif‐1α is knocked down in enterocytes using RNAi, but enhanced in flies where Hif‐1α is overexpressed. It is probable that superoxide from site III_Qo_ is elevated in enterocytes in flies reared on 5% YE, which stabilizes Hif‐1α, which in turn contributes to an increase in intestinal permeability and decrease in lifespan which can be ameliorated by S3QELs.

**FIGURE 5:**
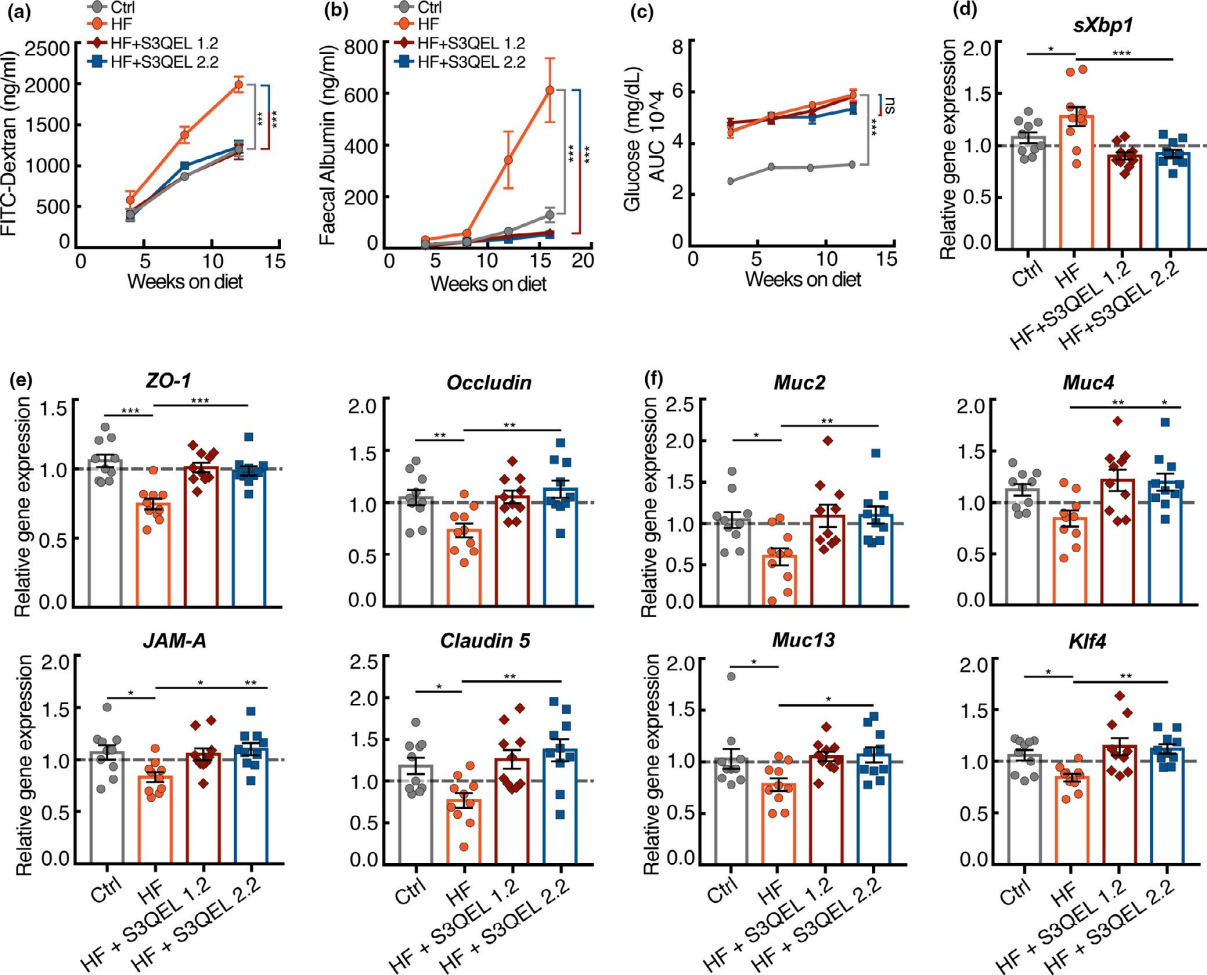
Effects of S3QELs on intestinal parameters and blood glucose levels in C57BL/6J mice fed a high‐fat diet. Male C57BL/6J mice were fed chow (Ctrl) or high‐fat (HF) diet ±200 mg/kg S3QELs, for 16 weeks. Effects of S3QELs on (a) plasma FITC‐Dextran after oral gavage and (b) albumin in fecal matter. (c) Effect of S3QELs on glucose tolerance (individual time point glucose tolerance curves are shown in Figure S9). (d, e, f) Effect of S3QELs on colonic expression of (d) an ER stress gene, (e) tight junction genes, and (f) mucin genes, normalized to β‐actin then expressed as fold change relative to control diet‐fed mice. (c) Data are means ± SEM of *n* = 10 mice. (a, b) ***p < 0.0001 by ANCOVA. (d, e, f) *p < 0.05, **p < 0.009, ***<0.0003 by one‐way ANOVA with Tukey's post‐test. (c) ***p < 0.0001 by two‐way ANOVA with Dunnett's multiple comparison. ns, not significant. AUC, area under the curve

To examine whether the effects of S3QELs in *Drosophila* are conserved, we tested whether they protect against intestinal permeability in a mouse model. To draw parallels to *Drosophila*, we tested oral delivery of S3QELs in male C57BL/6J mice fed a high‐fat diet (60% kcal). High‐fat feeding has been shown to increase oxidative stress and induce intestinal permeability in mice (Ahmad et al., 2017; Murakami et al., 2016). We found that high‐fat feeding significantly induced intestinal permeability in mice measured both by the uptake of FITC‐dextran from the gut into blood plasma (*F* = 48.64, *p* = 3.82E−09) (Figure 5a) and by the appearance of plasma‐derived albumin in feces (*F* = 22.74, *p* = 8.75E−06) (Figure 5b). In conjunction, there was decreased expression of tight junction (Figure 5e) and mucin genes (Figure 5f). It also induced glucose intolerance (Figure 5c; Figure S9A), increased body weight and adiposity (Figure S9B,C) and increased the expression of an ER stress gene (Figure 5d).

S3QEL1.2 and S3QEL2.2 strongly protected against the increases in intestinal permeability in mice by decreasing both plasma FITC‐dextran (*F* = 39.795, *p* = 8.78E−14) and fecal albumin (*F* = 37.913, *p* = 4.39E−14) (Figure 5a,b). They protected against the decrease in tight junction and mucin gene expression in both colon and distal small intestine (Figure 5e,d; Figure S8). Expression of the goblet cell differentiation transcription factor Klf4 is known to be decreased upon high‐fat feeding, and this decrease is one cause of decreased mucin expression (Gulhane et al., 2016). Klf4 expression was decreased by high‐fat feeding and protected by S3QELs (Figure 5f). Together, these results strongly suggest that superoxide production from mitochondrial complex III drives intestinal permeability in mice as it does in *Drosophila*. This effect was probably not systemic but through direct exposure of enterocytes to S3QELs from the gut lumen, since unbound plasma concentrations of S3QELs measured in morning‐drawn cardiac puncture blood were at least 1000× lower than their IC_50_ for suppression of superoxide production by isolated muscle mitochondria (Orr et al., 2015).

Weight gain (Figure S9B), and food consumption (Figure S9C) of mice fed the high‐fat diet were not significantly altered by S3QEL feeding. Treatment with S3QELs did not improve glucose tolerance in mice fed the high‐fat diet (Figure 5c; Figure S9A). Hyperglycemia induced by a high‐fat diet has been proposed to drive intestinal permeability (Thaiss et al., 2018). The lack of significant effect of S3QELs on glucose tolerance despite the improvement in intestinal permeability suggests that hyperglycemia is upstream of intestinal permeability and supports this model (Thaiss et al., 2018) over the alternative model that increased gut permeability drives glucose intolerance. We propose that high‐fat diet and the resulting hyperglycemia increase superoxide production from site III_Qo_ of the mitochondrial electron transport chain in gut epithelial cells, which in turn drives ER stress, increased intestinal permeability, and associated sequelae. This hypothesis explains why treatment with S3QELs protects against ER stress (Figure 5d) and intestinal permeability (Figure 5a,b), but not against impaired glucose tolerance (Figure 5c; Figure S9A).

## 2 DISCUSSION

There is an emerging realization that intestinal barrier dysfunction contributes to almost every major disease, including aging, and it has become critically important to understand the mechanisms that drive this dysfunction (Farhadi et al., 2003; Fasano & Shea‐Donohue, 2005; König et al., 2016; Odenwald & Turner, 2017). Oxidative stress has consistently been argued to be an important driver of aging and age‐related pathologies, including intestinal barrier dysfunction (Hale et al., 2012; Liguori et al., 2018; Rera et al., 2012; Tian et al., 2017; Wang et al., 2014). However, the sources of oxidative stress are often not addressed and their relative importance in a given pathology remains unclear. There are few examples connecting mitochondrial superoxide/hydrogen peroxide with intestinal barrier dysfunction. Prior studies report that mitochondrially targeted general antioxidants such as MitoQ protect against intestinal barrier disruption (Hale et al., 2012; Wang et al., 2014). However, they used a dextran sulfate sodium‐induced colitis mouse model (DSS model), which is an unphysiological “sledge‐hammer” approach. We utilized a diet‐induced barrier disruption model, which is more physiologically relevant to human aging and disease, and we used S1QELs and S3QELs to identify the source of the superoxide that causes intestinal barrier disruption.

It is becoming increasingly apparent that the site of superoxide/hydrogen peroxide production is important when understanding and treating pathology given that general antioxidants often have no benefit or have detrimental side effects (Bjelakovic et al., 2007). General antioxidants act as a sponge “mopping” up ROS in a non‐specific manner, which interferes with potentially important oxidative signaling necessary to normal physiology. The identification of S1QELs, which are specific small‐molecule Suppressors of site I_Q_ Electron Leak, and S3QELs, which are specific small‐molecule Suppressors of site III_Qo_ Electron Leak, offers precise tools to identify and prevent superoxide/hydrogen peroxide production by complex I or III and its downstream effects without interfering with other sites (Brand et al., 2016; Orr et al., 2015). Studies using these compounds have established that sites I_Q_ and III_Qo_ not only have the highest capacity of all mitochondrial sites to produce superoxide/hydrogen peroxide in vitro, but also that they are the main contributors of superoxide in the mitochondrial matrix in several cell lines (Brand, 2016; Fang et al., 2020; Wong et al., 2019). These tools enable investigation of the contributions and importance of superoxide/hydrogen peroxide production by mitochondrial sites I_Q_ and III_Qo_ in pathologies and physiology (Brand, 2020; Watson et al., 2019).

Understanding the mechanisms that drive intestinal barrier dysfunction is crucial to understanding the impact and connection of this barrier to both healthspan and lifespan. Three interrelated candidate mechanisms are ER stress (Gulhane et al., 2016) and hyperglycemia (Thaiss et al., 2018), both known to induce mitochondrial ROS production, and stabilization of Hif‐1α in response to superoxide/hydrogen peroxide produced from site III_Qo_ (Bell et al., 2007; Brunelle et al., 2005; Guzy et al., 2005). Our work builds mechanistically upon these earlier studies to show that superoxide produced specifically by site III_Qo_ of mitochondrial complex III is a crucial cause of the downstream damage and signaling caused by hyperglycemia and ER stress that leads to pathology.

We conclude that superoxide production from site III_Qo_ inferred from use of S3QELs contributes to the development of diet‐induced intestinal barrier dysfunction in flies and mice. In flies, raising superoxide levels by *Sod* knockdown in enterocytes is sufficient to cause intestinal barrier dysfunction. Increased diet‐induced intestinal permeability tightly correlates with decreased lifespan, and feeding S3QELs extends diet‐compromised lifespan in flies. This is important, as our study makes the first robust link from superoxide produced by mitochondrial complex III to the intestinal pathology that impacts lifespan, with important ramifications for mechanisms of metabolic disease and aging. Suppressing superoxide production by site III_Qo_ in complex III of the mitochondrial electron transport chain using S3QELs has potential therapeutic value in intestinal disorders and premature aging caused by overnutrition.

## CONFLICT OF INTERESTS

The authors declare no competing interests.

## AUTHOR CONTRIBUTIONS

M.A.W., P.K and M.D.B. designed the experiments, M.A.W. performed the experiments and wrote the manuscript; P.K and M.D.B. edited the manuscript. B.P. helped perform succinate feeding and Hif‐1α fly experiments. T.A.U.H. helped with statistical analysis, and J. L‐D. gavaged the mice.

## Supporting information

Fig S1‐S10

## Data Availability

All data is available in the manuscript or the supplementary materials.
