# Supplementary material for: S3QELs protect against diet‐induced intestinal barrier dysfunction
Source: Aging Cell. 2021 Sep 14;20(10):e13476. doi: 10.1111/acel.13476 (PMC8520719; doi:10.1111/acel.13476)
Supplement: Supplementary file 1 — Fig S1‐S10 [file ACEL-20-e13476-s001.docx]

**Supplementary Materials:**

**Methods and Materials**

#### *Experimental design*: Experiments were designed to examine the effects of suppressing the production of superoxide by complex III using S3QELs on intestinal permeability in *Drosophila* and mice.

#### *Fly strains and husbandry:* Control lines were *w^1118^* and *Canton S*. The GAL4 driver line was NP1-GAL4;tub-GAL80ts (67067) from Bloomington Drosophila Stock Center. The UAS responder lines were Sod1-RNAi (v108307) and Sod2-RNAi (v42162) from Vienna Drosophila Resource Center (VDRC), as well as Hif-1α-RNAi (BL26207) and Hif-1α-overexpression (BL9582) from Bloomington Drosophila Stock Center. The *Gene-switch* GAL4 driver 5966(GS) was from Dr. Heinrich Jasper. All fly lines were maintained on standard yeast medium containing (w/v) 1.5% dry active yeast (Saf-instant), 5% sucrose, 0.46% agar, 8.5% cornmeal and 1% v/v acid mix (a 1:1 v/v mix of 83.6% w/v orthophosphoric acid and 10% w/v propionic acid) in distilled water. The mixture was boiled with continuous stirring for 10 min and allowed to cool, then the acid mix was stirred in. The yeast medium was poured into vials (10 ml/vial) or bottles (30 ml/bottle), allowed to cool, and stored at 4°C. Flies were maintained at 25°C and 60% humidity under a 12-h light/dark cycle except where stated otherwise.

Experimental media were identical to standard yeast medium except for the type of yeast extract (Baker’s yeast #212750 BactoTM Yeast Extract, B.D. Diagnost Systems, Sparks, MD) and the yeast content, at 0.5% w/v (dietary restriction), 2.5% (conventional), and 5.0% (*ad libitum*). S3QELs at different concentrations in the same volume of DMSO (a total of 0.04% v/v) were mixed into a cooled 5% yeast extract medium to give final concentrations of 0.08, 0.8, 8.0, and 80 μM, using DMSO as the vehicle control. S1QELs were added in the same way using only a final concentration of 8.0 μM. Where indicated, 50 mM dimethylsuccinate was added to 5.0% YE medium. As a control an equimolar amount of methanol was used assuming complete conversion of dimethylsuccinate when taken up. Hif-1α knockdown or overexpression flies were reared on either 0.5% YE or 5.0% YE containing either 100 uM RU486 dissolved in 95% ethanol (denoted as RU486+), or 0.5% YE or 5.0% YE containing the same volume of 95% ethanol (denoted as RU486-).

For crosses, 15 virgin females were crossed with 5 male flies in a stock bottle for 5 d, and then removed. Larvae were left to develop until eclosion, then flies were transferred to a fresh bottle and aged for 5 d to allow the intestine to mature. Female flies were then sorted under CO_2_ anesthesia into experimental vials containing 20-25 flies. For *Sod1* and *Sod2* knockdown using NP1-GAL4;tub-GAL80ts, flies were crossed and maintained in an incubator at 18°C. Once eclosed, flies were aged for 5 d, sorted onto experimental diets, then maintained in an incubator at 29°C to induce knockdown.

***Lifespan Analysis:*** Flies were crossed on standard yeast medium. After eclosion, progeny were transferred to a fresh bottle containing the same medium and aged for 5 d. Mated females were then transferred to experimental diets. For lifespan analysis, 8 vials containing 25 flies were sorted giving a total of 200 flies per condition. Flies were transferred to a fresh vial every other day at which time the numbers of live and dead flies were recorded on an Excel spreadsheet. Lifespan was analyzed in Prism 7 using a Kaplan-Meir analysis to determine median lifespan and a log-rank test to determine statistical significance.

***Fly intestinal permeability:*** Flies that were assayed for lifespan were simultaneously assayed for intestinal permeability as previously described^15^. Every 5-10 days flies were transferred to their equivalent experimental diet but containing 2.5% w/v blue food coloring (FD&C #1). After 24 h intestinal permeability was scored on a yes/no basis by the appearance of blue dye in the hemolymph, which made their entire body appear blue. Intestinal permeability is presented as % blue flies within each experimental group. Intestinal permeability was calculated as: ^n^Smurfs/(^n^Smurfs + ^n^Non-Smurfs) x 100.

***Acridine Orange/Ethidium Bromide staining for intestinal apoptosis:*** 12 fly intestines per condition and experimental replicate were dissected into 1X phosphate buffered saline (PBS). Intestines were stained with 5 μg/ml of a 1:1 mix by weight of Acridine Orange/Ethidium Bromide for 5 min, washed 3 times for 10 min with 1X PBS, fixed with 4% v/v paraformaldehyde (PFA) in 1X PBS for 20 min, washed 3 times for 10 min with 1X PBS, then incubated with DAPI for 15 min, and mounted in mowiol mounting medium on a microscope slide. Apoptotic cells were counted from the hindgut-posterior midgut intersection up to the proventriculus using an Olympus BX51 fluorescence microscope immediately after mounting.

***Phosphohistone H3 (PH3) staining for proliferating cells:*** 12 fly intestines per condition and experimental replicate were dissected into 1X PBS then fixed with 4% v/v paraformaldehyde in 1X PBS for 45 min, washed for 1 h with wash buffer (1X PBS, 0.5% w/v fatty acid-free bovine serum albumin, and 0.1% v/v Triton X-100), then incubated with rabbit anti-PH3 primary antibody (1:1000, Millipore) in wash buffer overnight at 4°C. The primary antibody was removed and the intestine was washed with wash buffer for 1 h at 4°C, incubated with anti-rabbit Alexa fluor 555 secondary antibody for 4 h at room temperature, washed with wash buffer, stained with DAPI for 15 min, then mounted in mowiol mounting medium on a microscope slide. PH3-positive cells were counted from the hindgut-posterior midgut intersection up to the proventriculus using an Olympus BX51 fluorescence microscope.

***Fly food consumption:*** Food consumption was determined as previously described^45^. Flies maintained on experimental diets for 10 d were transferred to vials containing their equivalent experimental diet but with added 2.5% w/v blue food coloring (FD&C #1) 1 h after the lights came on in the morning. After 15 min, and 4 h, flies were snap-frozen in liquid nitrogen. Five flies were then homogenized in 100 μL 1X PBS containing 1% v/v Triton X-100. The amount of blue food coloring present in the homogenate was determined by measuring the absorbance at 630 nM.

***Mice:*** Six-week-old male C57BL/6J mice were purchased from Jackson Laboratories and acclimated in-house for two weeks before the study. At 8 weeks of age, mice were randomized into groups based on weight and fed either a control semi-purified diet D12450J containing 10% fat kcal, a 60% fat kcal diet D12492, or a custom-mixed 60% fat kcal diet D12492 containing 200 mg/kg of S3QELs. Diets were purchased from Research Diets and were gamma-irradiated. Mice were maintained on the experimental diets for 16 weeks in total. Food consumption was measured daily in a semiquantitative fashion. The food was weighed before placing in the cage, and then weighed again 24 h later. The total food weight difference within 24 h was divided by the number of mice in the cage to determine average food consumption per mouse. All experiments involving the use of mice were reviewed and approved by the Institutional Animal Care and Use Committee (IACUC) of the Buck Institute.

***FITC-dextran:*** Mice were fasted for 4 h prior to assay. Fluorescein isothiocyanate–dextran (FITC-Dextran) molecular weight 4000 (Sigma Aldrich) was administered by oral gavage using a needle attached to a 1 ml syringe at 600 mg/kg body weight. 4 h post FITC-dextran gavaging ~100 μL of blood was collected via tail bleeding into an EDTA-coated Eppendorf tube and placed on ice. Blood samples were centrifuged for 10 min at 5000 g to isolate blood plasma. Blood plasma samples were diluted appropriately using 1X PBS and FITC-dextran fluorescence was measured at Ex 485 nm/Em 535 nm. FITC-dextran amount was determined using standard curves generated from a known amount of FITC-dextran titrated in plasma from control or high fat-fed mice.

***Fecal Albumin:*** Feces were collected from individual mice by hand restraining and allowing the mouse to excrete directly into a 1-mL Eppendorf tube. Samples were kept on ice. At assay, fecal samples were diluted to 10 and 100 mg/mL using 1X PBS then centrifuged at 10,000 g for 3 min to pellet fecal debris. 100 μL of the supernatant was assayed to determine albumin concentration using a mouse albumin ELISA (Bethyl Laboratories) according to the manufacturer’s guidelines.

***Glucose tolerance:*** Mice were fasted for 6 h then a baseline blood glucose concentration was determined before intraperitoneal injection of glucose at 2 mg/kg body weight using an insulin syringe. Blood glucose was measured 15, 30, 45, 60, 90, and 120 min after glucose injection using an Accucheck Aviva glucometer.

***Total RNA and cDNA preparation:*** 15 fly intestines from each experimental group were dissected (Malpighian tubules removed) and placed into 200 μL of RNA lysis buffer. Fly intestines were manually homogenized using a Kontes microtube pellet pestle rod.

Approximately 50-100 mg of frozen mouse colon or small distal intestine from 10 mice from each experimental group was individually added to 1 mL of TRIzol (Invitrogen) with a stainless steel bead. Intestinal samples were disrupted using a Qiagen TissueLyser Bead Homogenizer for 8 min at 20 Hz. 0.2 mL of chloroform was added, vortexed, left to incubate for 2-3 min, then centrifuged at 12,000 g for 15 min.

RNA from the fly intestine lysates, and the aqueous phase from the mouse intestinal Trizol lysates, were further processed to isolate RNA using a Quick-RNA™ MiniPrep Kit (Zymo Research) according to the manufacturer’s guidelines. RNA from the Zymo-spin IIICG column was eluted using 30 μL of DNAse/RNAse-free water by centrifugation for 1 min at 12,000 g. The quantity and quality of fly and mouse RNA samples were determined using a NanoDrop 1000 spectrophotometer (Thermo Scientific). 1 μg of total RNA from either fly or mouse intestine was used in a total reaction volume of 20 μL containing 4 μL of iScript Reverse Transcription Supermix (Bio-Rad) to synthesize cDNA according to the manufacturer’s guidelines. cDNA was stored at -20°C before carrying out quantitative RTPCR.

***Quantitative real-time PCR (qPCR)*:** A 10 μL reaction volume containing 200 ng of cDNA, 400 nM of forward and reverse primer and 5 μL of SensiFAST SYBR No-ROX Kit (BIOLINE) was used in a qPCR reaction performed using a Light Cycler 480 Real-Time PCR System (Roche Applied Science). For the fly intestinal samples, fold changes in gene expression were determined using the 2^(-ΔΔCt) method. Gene expression was normalized to RpL32. For the mouse intestinal samples, the mean ΔCt value for the control group was determined, and the control mouse ΔCt value closest to the mean of the entire group was used as the calibrator to determine gene expression fold change using the 2^(-ΔΔCt) method. Gene expression was normalized to β-actin.

***Statistical Analysis*:**

Data are means ± SEM. Differences between groups were analyzed by either one-way ANOVA with Tukey’s test, two-way ANOVA with Dunnett’s multiple comparison test, three-way ANOVA, or ANCOVA as appropriate. Lifespans were analyzed by either LogRank (Mantel-Cox) or Cox proportional hazard GLM test. Statistics were analyzed and graphs were generated using Microsoft Excel, GraphPad Prism and R.

***Primer Lists:***

**Drosophila**

| **Primer name** | **Primer Sequence** |
| --- | --- |
| RpL32 5’ | CAACAGAGTCGGTCGCCGCTTC |
| RpL32 3’ | CAGCTCGCGCACGTTGTGCACC |
| Dpt 5’ | CACGAGATTGGACTGAATGG |
| Dpt 3’ | TTTCCAGCTCGGTTCTGAGT |
| Drs 5’ | GAGGAGGGACGCTCCAGT |
| Drs 3’ | TTAGCATCCTTCGCACCAG |
| Def 5’ | TTTTGCTCTGCTTGCTTGC |
| Def 3’ | ACATGATCCTCTGGAATTGGA |
| upd3 5’ | ACCTACAGAAGCGTTCCAG |
| upd3 3’ | GGTTCTGTAGATTCTGCAGG |
| Tsp2A 5’ | GTAATGATGGCCGTCAGCTT |
| Tsp2A 3’ | TGGTCAGCACGTAGTTCGAG |
| Ssk 5’ | TTCGGCACAACCAAACATAAG |
| Ssk 3’ | GTGGTTCGCACAGCTCTCT |
| mesh 5’ | ACCAAAAGCGGTTGACATTC |
| mesh 3’ | CAGGGAATTCAGCTGGGATA |
| Bbg 5’ | GTGGTGTCAACGATGTCCT |
| Bbg 3’ | ACCGCAGTACGGTGAATAGG |
| Cora 5’ | GGGTCCACCTATCGCTACAA |
| Cora 3’ | CAGAGCTGAAAGGATCAGGT |
| Pyd 5’ | CACCACCTCGTCGTATTCCT |
| Pyd 3’ | GACTGCCGTACAACCAGGAT |
| Crb 5’ | CAGGAGCAGCAATCTGACGA |
| Crb 3’ | GGTCACTCGTCCTCCGTTTA |
| Baz 5’ | ATCATGCTGGTCATGGTCAA |
| Baz 3’ | GTCCGGCAGAGCAGTTAGT |

**Mouse**

| **Primer name** | **Primer Sequence** |
| --- | --- |
| β-Actin 5’ | AGCACTGTGTTGGCATAGAGGTC |
| β-Actin 3’ | CTTCTTGGGTATGGAATCCTGTG |
| ZO-1 5’ | CCACCTCTGTCCAGCTCTTC |
| ZO-1 3’ | CACCGGAGTGATGGTTTTCT |
| Occludin 5’ | TTGAAAGTCCACCTCCTTACAGA |
| Occludin 3’ | CCGGATAAAAAGAGTACGCTGG |
| JAM-A 5’ | TCTCTTCACGTCTATGATCCTGG |
| JAM-A 3’ | TTTGATGGACTCGTTCTCGGG |
| Claudin 5 5’ | GCAAGGTGTATGAATCTGTGCT |
| Claudin 5 3’ | GTCAAGGTAACAAAGAGTGCCA |
| Muc2 5’ | ATGCCCACCTCCTCAAAGAC |
| Muc2 3’ | GTAGTTTCCGTTGGAACAGTGA |
| Muc4 5’ | GCTCAAGTTGACAAGGAGCAGAGC |
| Muc4 3’ | GGAGGACAAAAGAAGGCGTGGCC |
| Muc13 5’ | GCCAGTCCTCCCACCACGGTA |
| Muc13 3’ | CTGGGACCTGTGCTTCCACCG |
| Klf4 5’ | AGCCACCCACACTTGTGACTATG |
| Klf4 3’ | CAGTGGTAAGGTTTCTCGCCTGTG |
| sXbp1 5’ | GAGTCCGCAGCAGGTGC |
| sXbp1 3’ | CAAAAGGATATCAGACTCAGAATCTGAA |

**
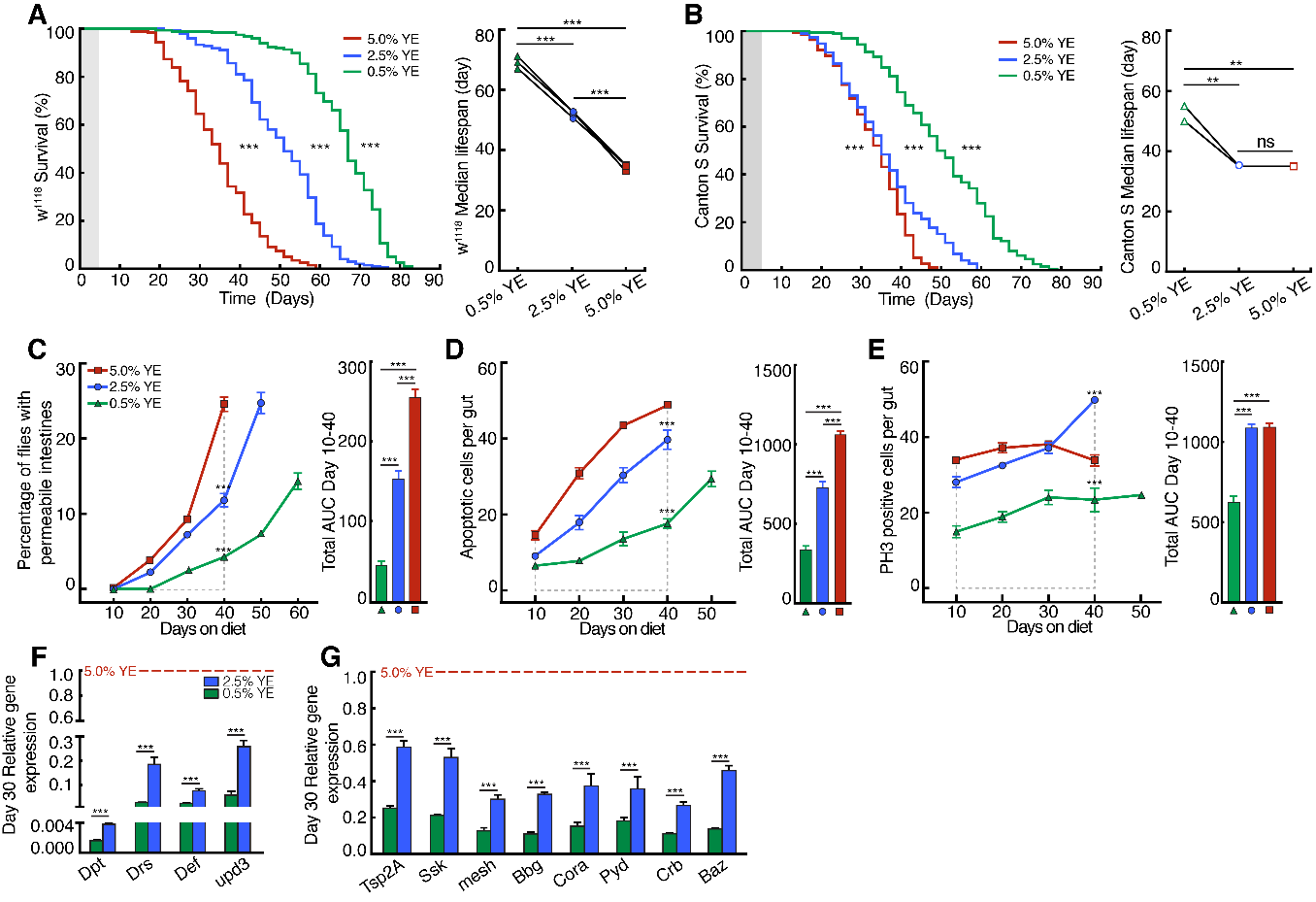
**

**Figure S1. Effects of diet on intestinal parameters and lifespan in *Drosophila.***

After eclosion *Drosophila* were raised for five days on standard yeast medium (shaded bars), then switched on day 5 to diets containing 0.5-5% YE. Effects of YE% on lifespan in **(A)** *w^1118^* and **(B)** *Canton S* flies and on (**C**) intestinal permeability, (**D**) intestinal apoptosis, and **(E)** intestinal stem cell proliferation in *Canton S* flies. (**F, G**) Day 30 intestinal gene expression on different YE% diets in *w^1118^* flies, normalized to *Rp49* and expressed as fold change relative to 5% YE. Panels show (**F**) inflammatory and damage markers and (**J**) intestinal tight-junction genes. Data are means ± SEM of n=3 biological replicates each using 200 flies (*A, B*), or 12 (*C-E*) or 15 (*F-G*) dissected intestines. (*A, B*) ***P<0.0001 by log-rank (Mantel-Cox) test for differences between lifespan curves. For median lifespan analysis ***P<0.0003, **P<0.005, *P<0.05, ns, not significant, by one-way ANOVA with Tukey’s post test. Shaded boxes in lifespan graphs (*A, B*) indicate the 5-day post eclosion period before flies were transferred to 5% YE. Dotted lines in (*C-E*) denote data points used for statistical analysis. (*C-G*) *P<0.05, ***P<0.0001 by one-way ANOVA with Tukey’s post-test. AUC, area under the curve.

**
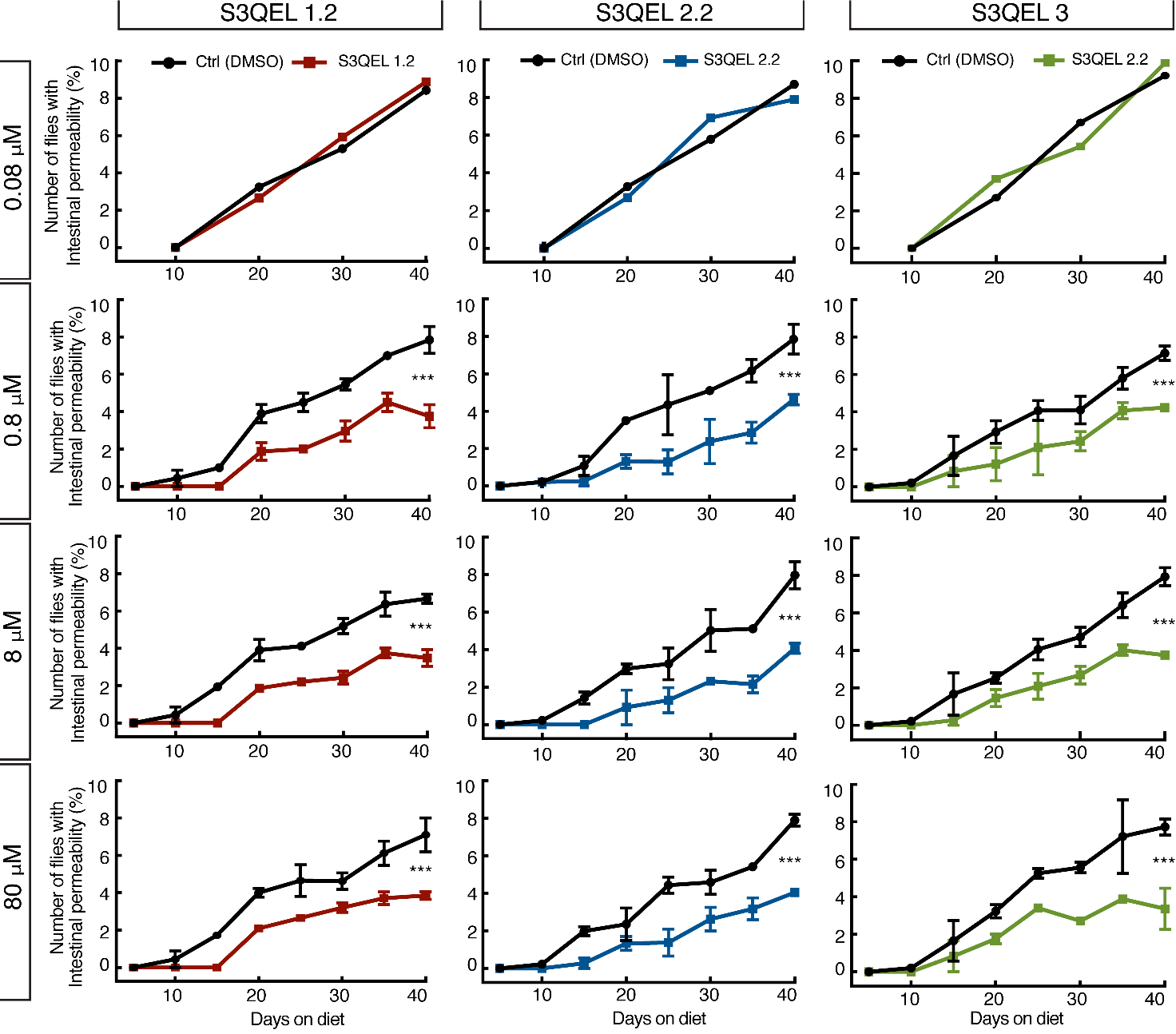
**

**Figure S2. Effect of S3QELs on intestinal permeability in *w^1118^* flies fed 5% YE.**

After eclosion *w^1118^* *Drosophila* were raised for five days on standard yeast medium, then switched on day 0 to a diet containing 5% YE with S3QEL or DMSO. Panels show the effects of three structurally different S3QELs at a range of concentrations on intestinal permeability. Data are means ± SEM of n=3 biological replicates each using 200 flies. ***P<0.0001 by two-way ANOVA with Dunnett’s multiple comparison.


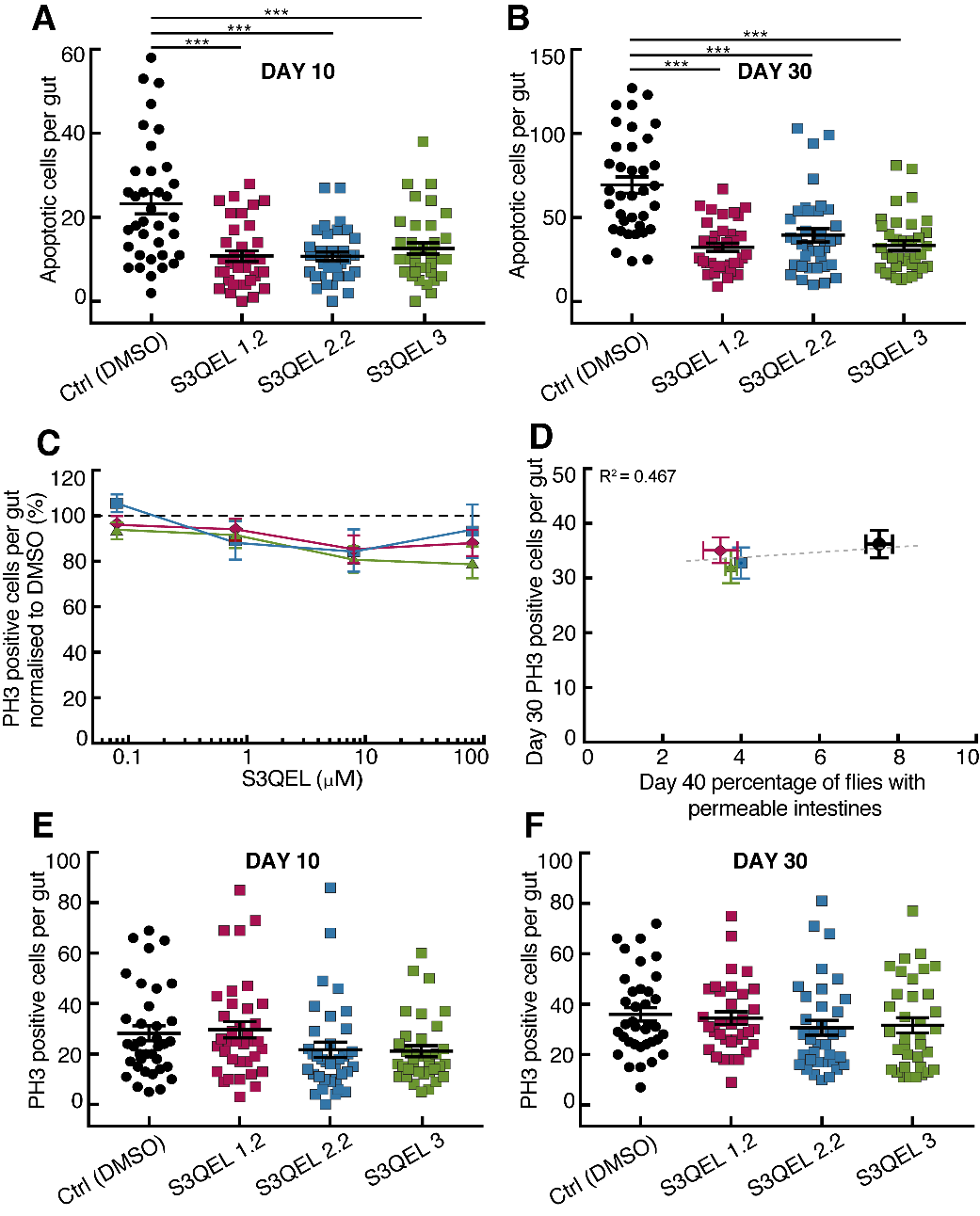


**Figure S3. Effects of S3QELs on intestinal apoptosis and proliferation on flies fed 5% YE.**

After eclosion *w^1118^* *Drosophila* were raised for five days on standard yeast medium, then switched on day 0 to diets containing 5% YE with S3QEL or DMSO. Effect of 8 μM of S3QELs on the number of intestinal apoptotic cells at (**A**) day 10, and (**B**) day 30. (**C**) Effect of S3QELs on the number of intestinal stem cells proliferating at day 30 as % of DMSO vehicle control. (**D**) Relationship between intestinal proliferation and intestinal permeability in 8 μM S3QEL- and DMSO-treated flies. The line in *D* was fit using linear regression. Effects of 8 μM of S3QELs on the number of intestinal stem cells proliferating at (**E**) day 10, and (**F)** day 30. Data are means ± SEM of n=3 biological replicates each using 12 intestines. ***P<0.0001 by one-way ANOVA with Tukey’s post-test.


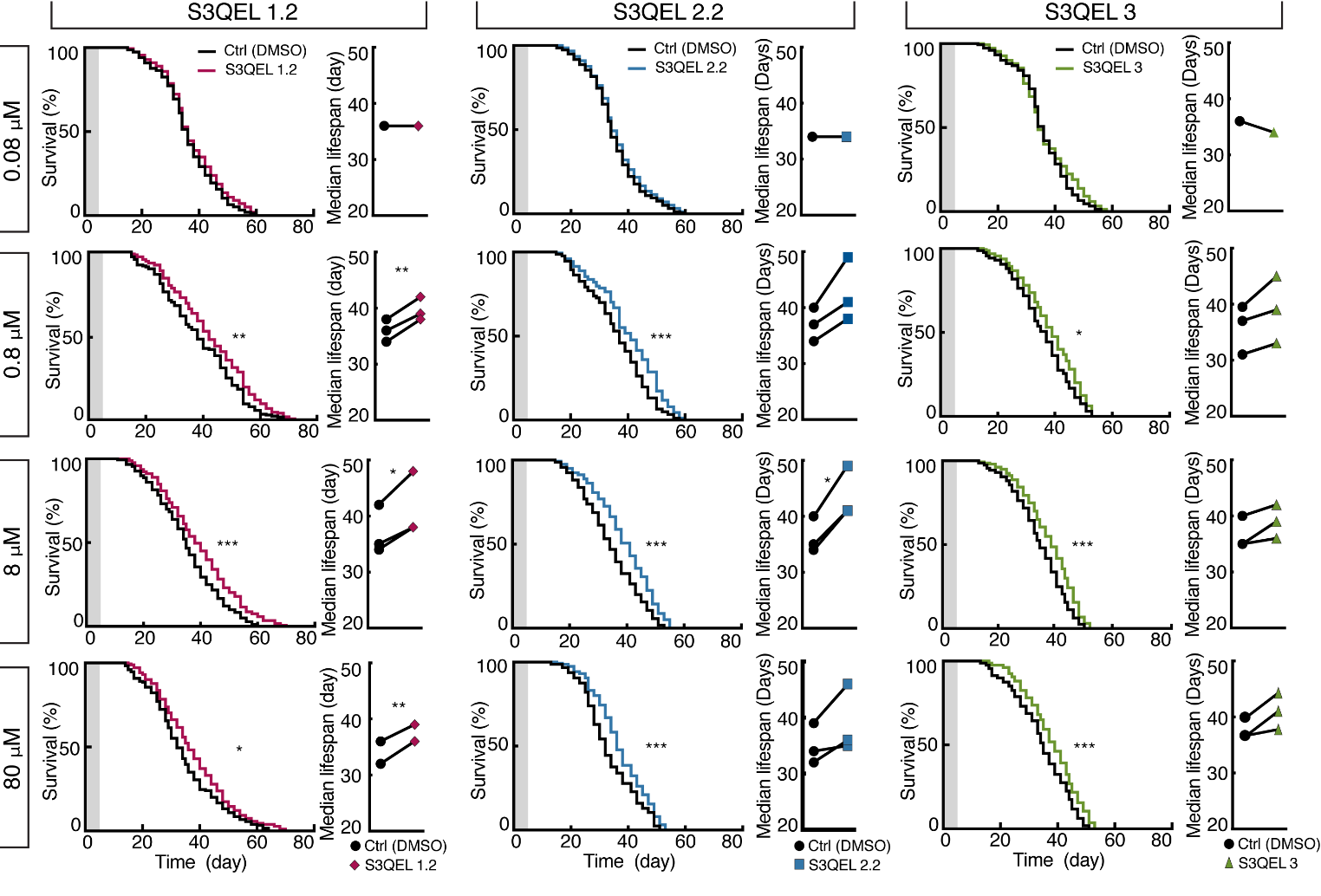


**Figure S4. Effect of S3QELs on lifespan in flies fed 5% YE.**

After eclosion *w^1118^* *Drosophila* were raised for five days on a standard yeast medium, then switched on day 5 to a diet containing 5% YE with S3QEL or DMSO. Panels show the effects of S3QELs at a range of different concentrations on lifespan. Shaded boxes in lifespan graphs indicate the 5-day post-eclosion period before flies were transferred to 5% YE and S3QEL treatment. Data are means ± SEM of n=3 biological replicates each using 200 flies. *P<0.05, **P<0.003, ***P<0.0001 by log-rank (Mantel-Cox) test to analyze lifespan curves. *P<0.05, **P<0.003 by paired *t*-test to analyze median lifespan.


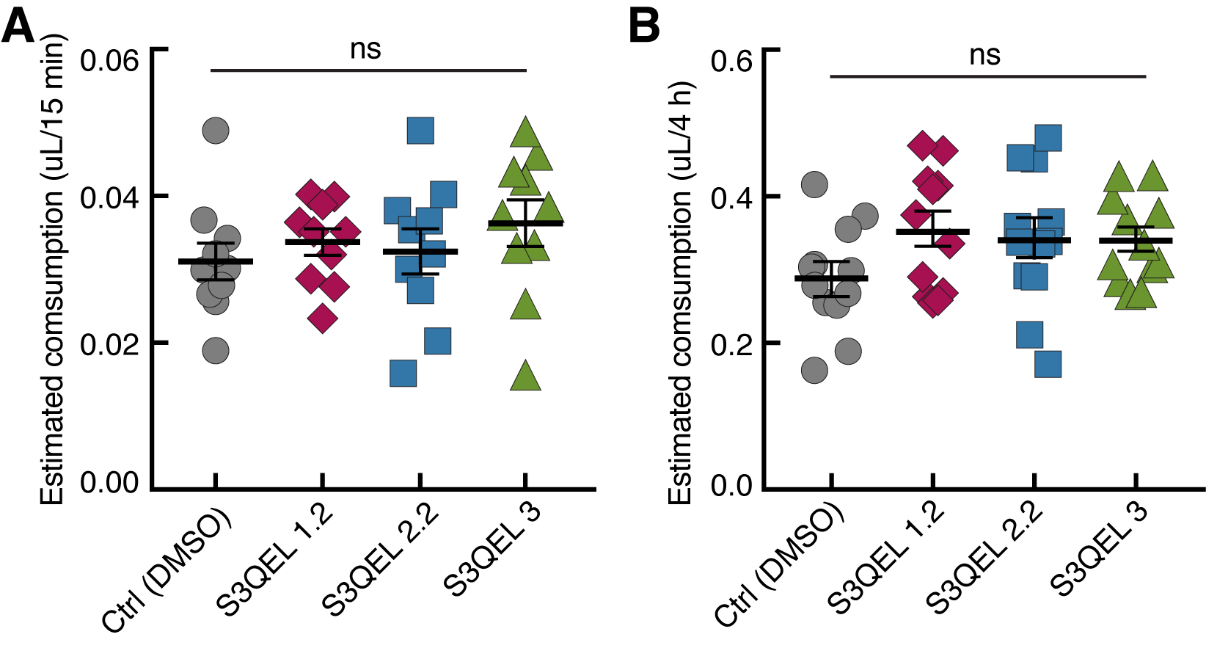


**Figure S5. Effect of S3QELs on food consumption of *W^1118^* flies fed 5% YE.**

Flies were raised on a 5% YE and either given DMSO as control or fed S3QELs at 8 μM. After 10 days of habituation on this food, food intake per fly was assayed by (**A**)15-min dye uptake and (B) 4-h dye uptake. Data are means ± SEM of n=10 samples (each point is the average of five homogenized flies). ns, not significant by one-way ANOVA with Tukey’s post-test.


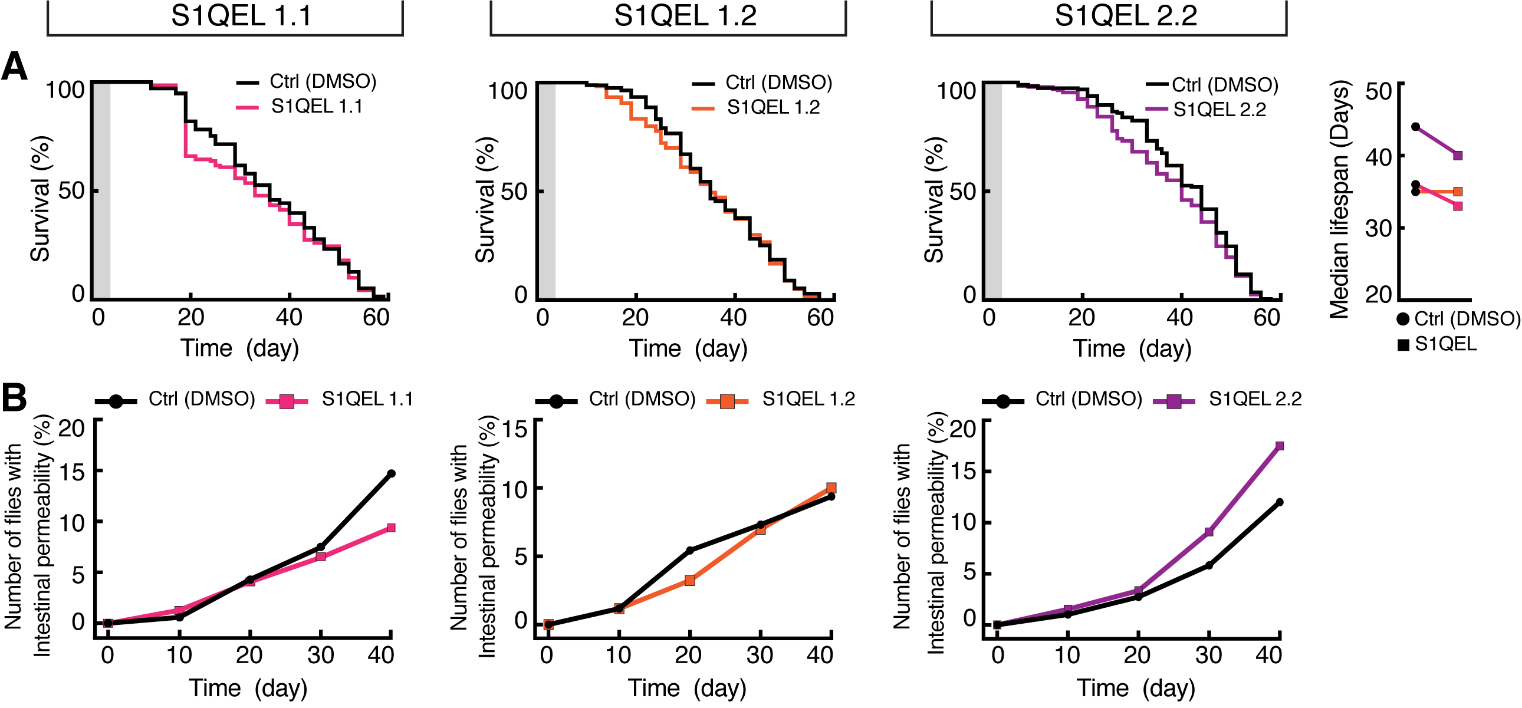


**Figure S6. Effect of S1QELs on lifespan and intestinal permeability in *w^1118^* flies fed on 5% YE.**

After eclosion *w^1118^* *Drosophila* were raised for five days on standard yeast medium, then switched on day 0 to a diet containing 5% YE with S1QEL or DMSO. Effect of three different S1QELs at a concentration of 8 μM on (**A**) lifespan, and (**B**) intestinal permeability. Data are n=1 biological replicate using 200 flies. Shaded boxes in lifespan graphs (**A**) indicate the 5-day post-eclosion period before flies were transferred to 5% YE.


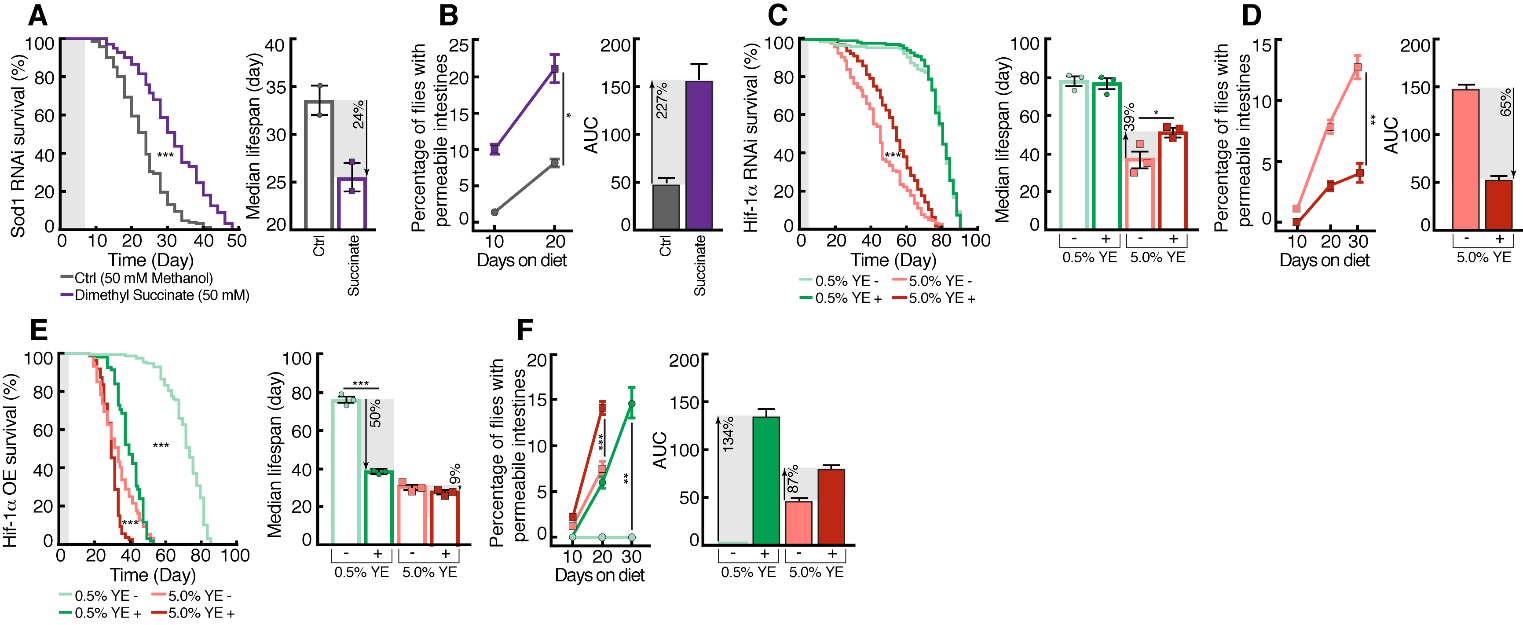


**Figure S7. Effects of succinate supplementation, and knockdown and overexpression of Hif-1α on lifespan and intestinal permeability in *w^1118^* flies fed either 0.5% YE or 5% YE.**

After eclosion *w^1118^* *Drosophila* were raised for five days on standard yeast medium, and then switched on day 0 to a diet containing 5% YE supplemented with either 50 mM methanol or 50 mM dimethylsuccinate. Effect of dimethylsuccinate supplementation on (**A**) lifespan, and (**B**) intestinal permeability. After eclosion *Drosophila* were raised for five days on standard yeast medium, and then switched on day 0 to a diet containing either 0.5% YE or 5% YE with RU486 (+) or without RU486 (-). The enterocyte Gal4 driver 5966-*GS* was used to knock down Hif-1α (sima); panels show the effect of Hif-1α knockdown on (**C**) lifespan, and (**D**) intestinal permeability. The same enterocyte Gal4 driver 5966-*GS* was used to overexpress Hif-1α (sima); panels show the effect of Hif-1α overexpression on (**E**) lifespan, and (**F**) intestinal permeability. (A,B) Data are n=2 biological replicates using 140 flies. (C,D,E,F) Data are n=3 biological replicates using 140 flies. Data are means ± SEM (or range where n=2). *P<0.05, **P<0.009, ***<0.0001 by unpaired *t*-test to measure median lifespan and AUC, log-rank (Mantel-Cox) test to analyze lifespan curves, or two-way ANOVA with Tukey’s post-test to analyze intestinal permeability. Shaded boxes in lifespan graphs (A,C,E) indicate the 5-day post-eclosion period before flies were transferred to 5% YE. AUC, area under the curve. -, without RU486. +, with RU486.


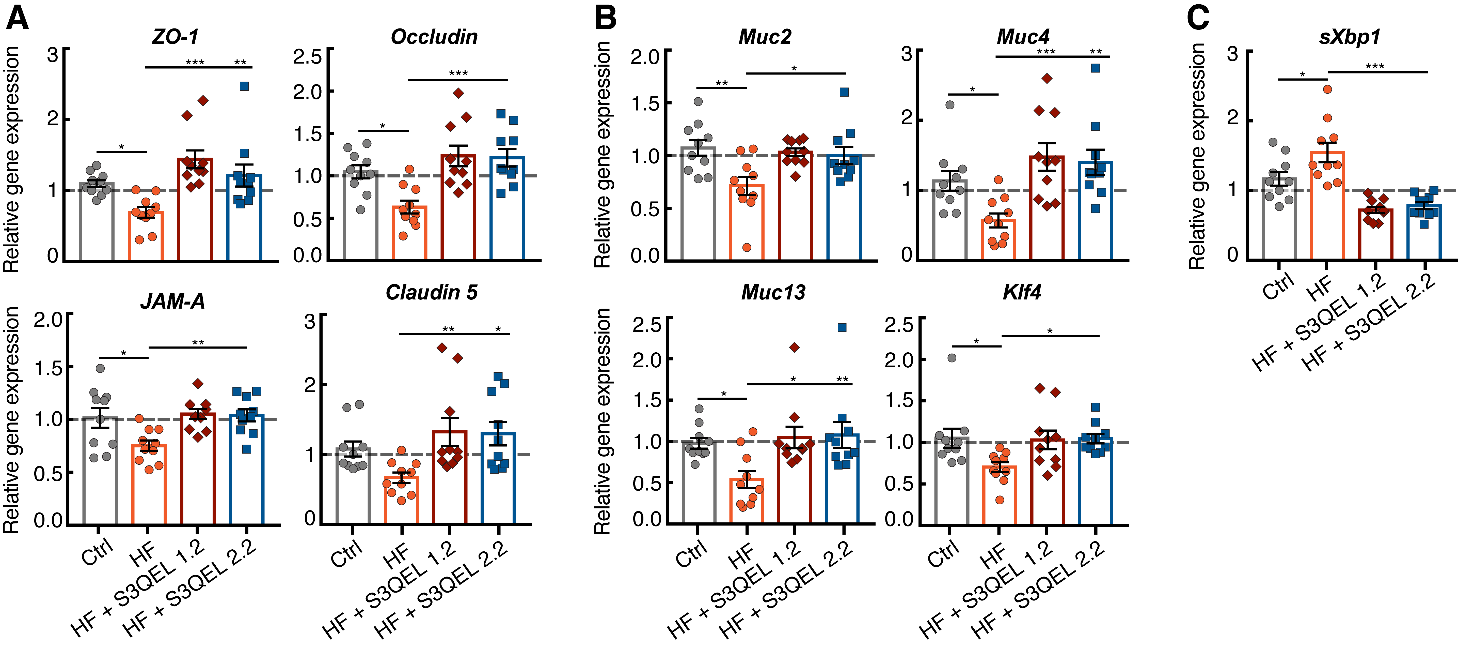


**Figure S8. Effect of S3QELs on gene expression in the small distal intestine in C57BL/6J mice fed a high-fat diet.**

Male C57BL/6J mice were fed chow (Ctrl) or high-fat (HF) diet ± 200 mg/kg S3QELs, for 16 weeks. Effect of S3QELs on colonic expression of (**A**) tight-junction genes, (**B**) mucin genes, and (**C**) an ER stress gene, normalized to β-actin then expressed as fold change relative to control diet-fed mice. Data are means ± SEM of n=10 mice. *P<0.05, **P<0.009, ***<0.0001 by one-way ANOVA with Tukey’s post-test.


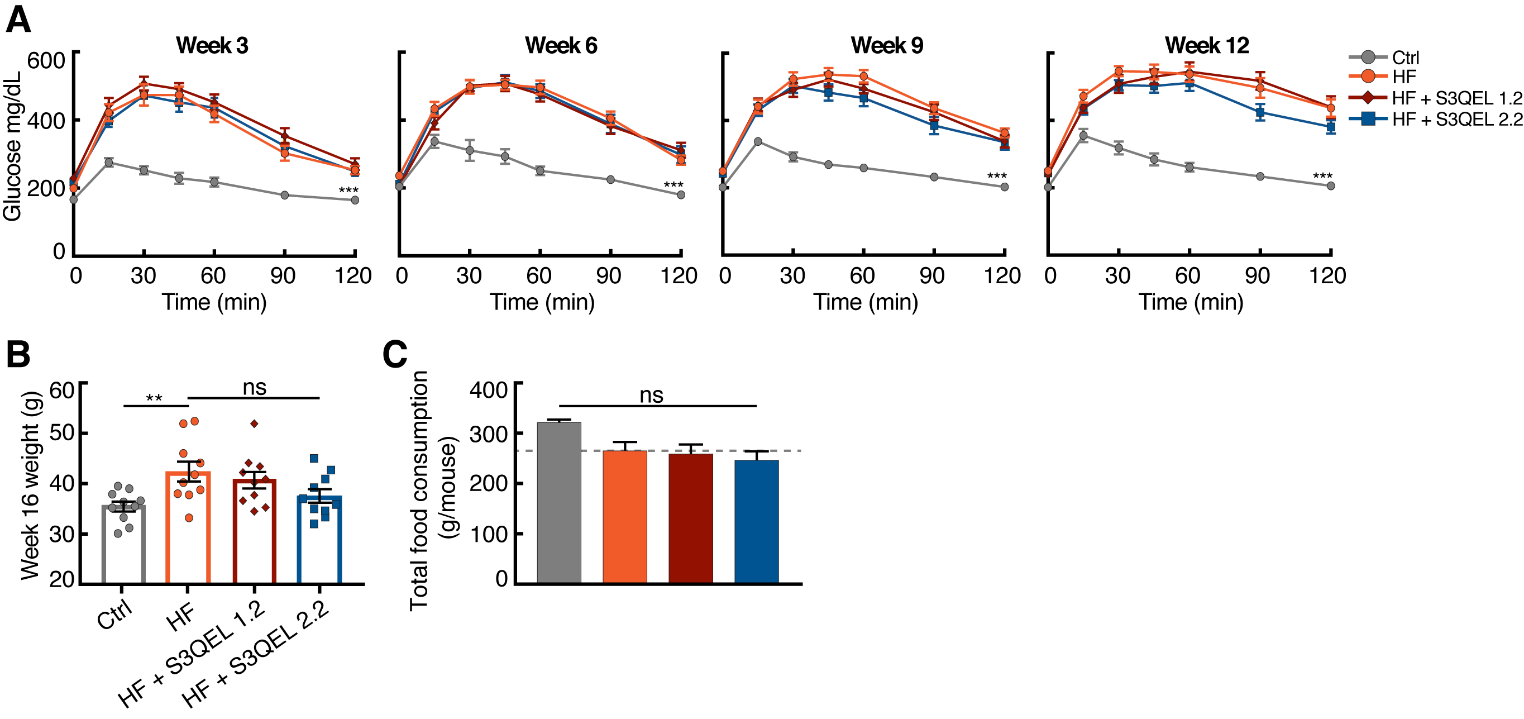


**Figure S9. Effect of S3QELs on metabolic measures in C57BL/6J mice fed a high-fat diet.**

Male C57BL/6J mice were fed chow (Ctrl) or high-fat (HF) diet ± 200 mg/kg S3QELs for 16 weeks. (**A**) Effect of S3QELs on glucose tolerance measured by the ability of each mouse to clear an intraperitoneal injection of 2 mg/kg glucose. Effect of S3QELs on (**B**) weight, and (**C**) food consumption over a 16-week period. Data are means ± SEM of n=10 mice. (*A*) ***P<0.0001, otherwise not significant, by two-way ANOVA with Dunnett’s multiple comparison. (*B*) *P<0.05, **P<0.003, ***<0.0001, ns, not significant, by one-way ANOVA with Tukey’s post-test.


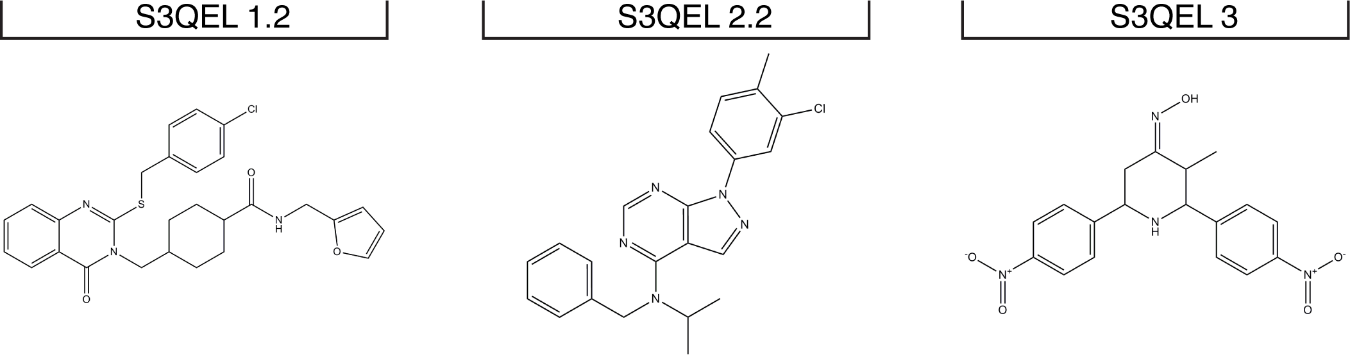


**Figure S10. Structures of the three different S3QELs used in this study**^28^

REFERENCE:

Deshpande, S. A., Carvalho, G. B., Amador, A., Phillips, A. M., Hoxha, S., Lizotte, K. J., & Ja, W. W. (2014). Quantifying Drosophila food intake: Comparative analysis of current methodology. Nature Methods, 11, 535–540.
